# Supplementary material for: Mutant KRAS promotes malignant pleural effusion formation
Source: Nat Commun. 2017 May 16;8:15205. doi: 10.1038/ncomms15205 (PMC5440809; doi:10.1038/ncomms15205)
Supplement: Supplementary Information — Supplementary Figures and Supplementary Tables [file ncomms15205-s1.pdf]

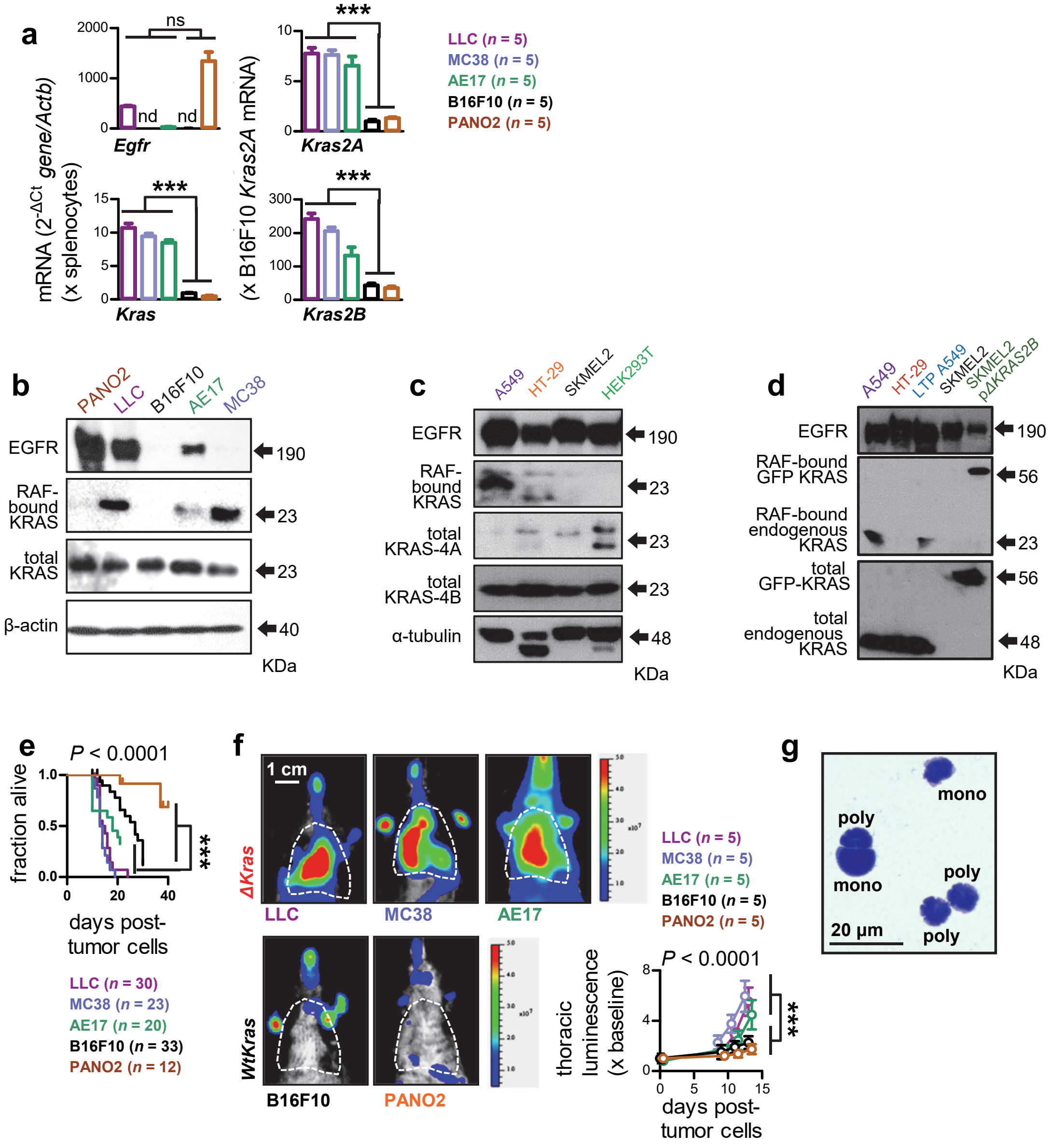

a
mRNA (2^-ΔCt gene/Actb) (x splenocytes)
Egfr
ns
nd
nd
(x B16F10 Kras2A mRNA)
Kras2A
***
Kras
***
Kras2B
***
LLC (n = 5)
MC38 (n = 5)
AE17 (n = 5)
B16F10 (n = 5)
PANO2 (n = 5)
b
PANO2
LLC
B16F10
AE17
MC38
EGFR
RAF-bound KRAS
total KRAS
β-actin
190
23
23
40
KDa
c
A549
HT-29
SKMEL2
HEK293T
EGFR
RAF-bound KRAS
total KRAS-4A
total KRAS-4B
α-tubulin
190
23
23
23
48
KDa
d
A549
HT-29
LTP A549
SKMEL2
SKMEL2 pΔKRAS2B
EGFR
RAF-bound GFP KRAS
RAF-bound endogenous KRAS
total GFP-KRAS
total endogenous KRAS
190
56
23
56
48
KDa
e
P < 0.0001
fraction alive
days post-tumor cells
***
LLC (n = 30)
MC38 (n = 23)
AE17 (n = 20)
B16F10 (n = 33)
PANO2 (n = 12)
f
1 cm
ΔKras
LLC
MC38
AE17
WtKras
B16F10
PANO2
LLC (n = 5)
MC38 (n = 5)
AE17 (n = 5)
B16F10 (n = 5)
PANO2 (n = 5)
P < 0.0001
thoracic luminescence (x baseline)
days post-tumor cells
***
g
mono
poly
mono
poly
poly
20 μm

Supplementary Figure 1 | *KRAS* expression patterns of mouse and human tumour cells and lethality and myeloid inflammatory response in malignant pleural effusions (MPE) induced by *Kras*-mutant murine tumour cells. a, Total *Kras*, *Kras* isoform A and B, and *Egfr* mRNA expression of five mouse tumour cell lines relative to *Actb* by qPCR (n = 4/group). Data are presented as mean ± SD. ns and ***: $P > 0.05$ and $P < 0.001$ for the comparison between any *Kras*-mutant and any *Kras*-wild-type cell line by one-way ANOVA with Bonferroni post-tests. **b-d,** EGFR, active KRAS, KRAS isoform, and total KRAS protein expression of mouse and human tumour cells relative to β-actin by immunobloting. **e,** Kaplan-Meier plot of the survival of *C57BL/6* mice after intrapleural injection of 1.5 x 10^5 mouse tumour cells. P: overall probability value; ***: $P < 0.001$ for the comparison between any *Kras*-mutant and any *Kras*-wild-type cell line by log-rank test. **f,** Representative bioluminescent images taken 14 days after pleural tumour cells and data summary of longitudinal chest light emission measurements from total body-irradiated *C57BL/6* chimeras transplanted with luminescent bone marrow from *CAG.Luc.eGFP* donors (n = 3/group/time-point). Data are presented as mean ± SD. P: overall probability value; ***: $P < 0.001$ for the comparison between any *Kras*-mutant and any *Kras*-wild-type cell line by two-way ANOVA with Bonferroni post-tests. **g,** Representative image of May-Gruenwald-Giemsa-stained CD11b+Gr1+ cells sorted by fluorescence-assisted cell sorting from a LLC-induced MPE, showing mixed polymorphonuclear (poly) and mononuclear (mono) morphology. *Wt*, wild-type; *Δ*, mutant; LLC, *C57BL/6* Lewis lung carcinoma; MC38, *C57BL/6* colon adenocarcinoma; AE17, *C57BL/6* malignant pleural mesothelioma; B16F10, *C57BL/6* malignant skin melanoma; PANO2, *C57BL/6* pancreatic adenocarcinoma; FULA, *FVB* urethane-induced lung adenocarcinoma; CULA, *C57BL/6* urethane-induced lung adenocarcinoma; A549, human lung adenocarcinoma; LTP A549, long-term passaged A549 cells having lost the Y chromosome; SKMEL2, human malignant skin melanoma; HT-29, human colon adenocarcinoma; HEK293T, human embryonic kidney cells.

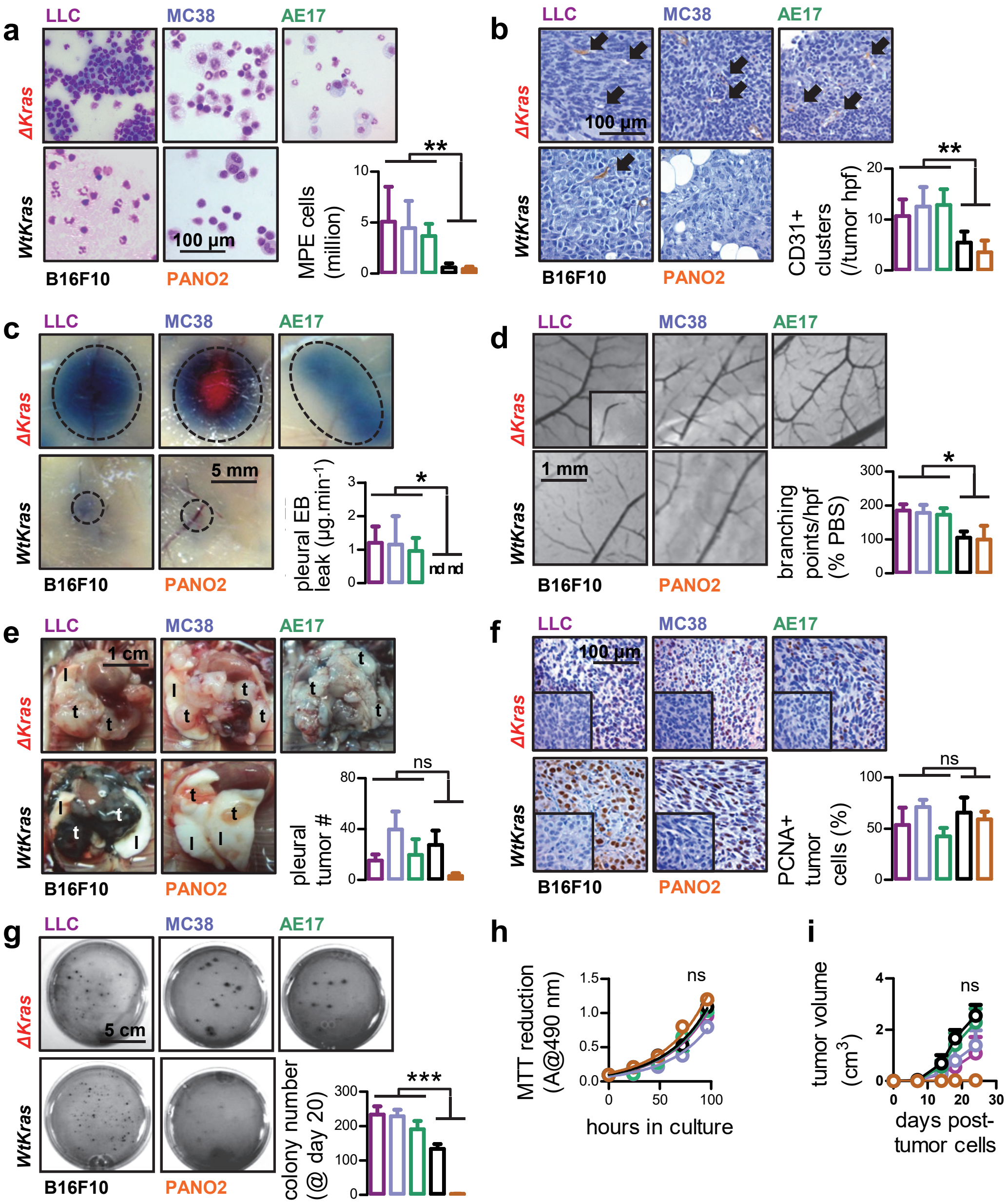

a
LLC
MC38
AE17
ΔKras
WtKras
100 μm
B16F10
PANO2
MPE cells (million)
**
b
100 μm
CD31+ clusters (/tumor hpf)
**
c
5 mm
pleural EB leak (μg.min-1)
*
nd nd
d
1 mm
branching points/hpf (% PBS)
*
e
1 cm
pleural tumor #
ns
f
100 μm
PCNA+ tumor cells (%)
ns
g
5 cm
colony number (@ day 20)
***
h
MTT reduction (A@490 nm)
hours in culture
ns
i
tumor volume (cm3)
days post-tumor cells
ns

Supplementary Figure 2 | Features of malignant pleural disease of *C57BL/6* mice induced by five syngeneic tumour cell lines: association of inflammation, angiogenesis, and vascular hyperpermeability with the presence of *Kras* mutations. a, b, e, f, 1.5 x 10^5 LLC, MC38, AE17, B16F10, or PANO2 tumour cells were delivered by direct pleural injection and the recipient *C57BL/6* mice (n = 10-15/group) were sacrificed after two weeks. Cell line colour codes are as in Extended Data Fig. 1a. **a,** Representative pleural fluid cytocentrifugal specimens (images) and total pleural fluid cell numbers (graph; *n* = 8-15/group). **b,** Representative microphotographs of pleural tumour tissue CD31 immunoreactivity (images) and pleural tumour microvessel (black arrows) density (graph; *n* = 10-15/group). **e,** Representative lung explants with lungs (l) and pleural tumours (t) (images) and pleural tumour foci abundance (graph; *n* = 10-15/group). **f,** Representative microphotographs of pleural tumour tissue proliferating cell nuclear antigen (PCNA) immunoreactivity (images) and pleural tumour cell proliferation index (graph; *n* = 5-10/group). **c,** Images: 50 µL cell-free MPE or pleural lavage supernatants obtained from experiment shown in (a, b, e, f) were injected intradermally into the shaved rear skin of *C57BL/6* mice (*n* = 5/group) followed by delivery of 0.8 mg intravenous albumin tracer Evans' blue. Mice were sacrificed and skins were inverted and imaged after 30 min for quantification of skin extravasation areas (dashed lines). Graph: 1.5 x 10^5 LLC, MC38, AE17, B16F10, or PANO2 tumour cells were delivered by direct pleural injection and the *C57BL/6* recipient mice (*n* = 5-12/group) received 0.8 mg intravenous albumin tracer Evans' blue two weeks later, and were sacrificed one hour thereafter. Shown are levels of bloodstream-to-pleural space extravasated albumin-bound Evans' blue. **d,** Phosphate-buffered saline (PBS) or 50 µL cell-free MPE or pleural lavage supernatants obtained from experiment shown in (a, b, e, f) were placed on fenestrated chick chorioallantoic membranes (*n* = 5/group) and membranes were imaged after five days. Shown are representative membrane vascular networks (images; insert is PBS control) and the increase in the number of branching points relative to PBS control (graph). **g,** 7,5 x 10^3 tumour cells were placed on soft agar-containing 60 mm Petri dishes (*n* = 5/group) and imaged after a month. Shown are representative colonies (images) and colony numbers (graph). **h,** 3 x 10^3 tumour cells were placed in DMEM-containing 96-well culture dishes (*n* = 5/group) and MTT reduction was monitored longitudinally. **I,** A million tumour cells were injected subcutaneously into the rear flank of *C57BL/6* mice (*n* = 5/group) and tumour dimensions were monitored longitudinally. Data are presented as mean ± SD. ns, *, ** and ***: $P > 0.05$, $P < 0.05$, $P < 0.01$, and $P < 0.001$ for the comparison between any *Kras*-mutant and any *Kras*-wild-type cell line by one (a-g)- or two (h and i)-way ANOVA with Bonferroni post-tests. LLC, *C57BL/6* Lewis lung carcinoma; MC38, *C57BL/6* colon adenocarcinoma; AE17, *C57BL/6* malignant pleural mesothelioma; B16F10, *C57BL/6* malignant skin melanoma; PANO2, *C57BL/6* pancreatic adenocarcinoma.

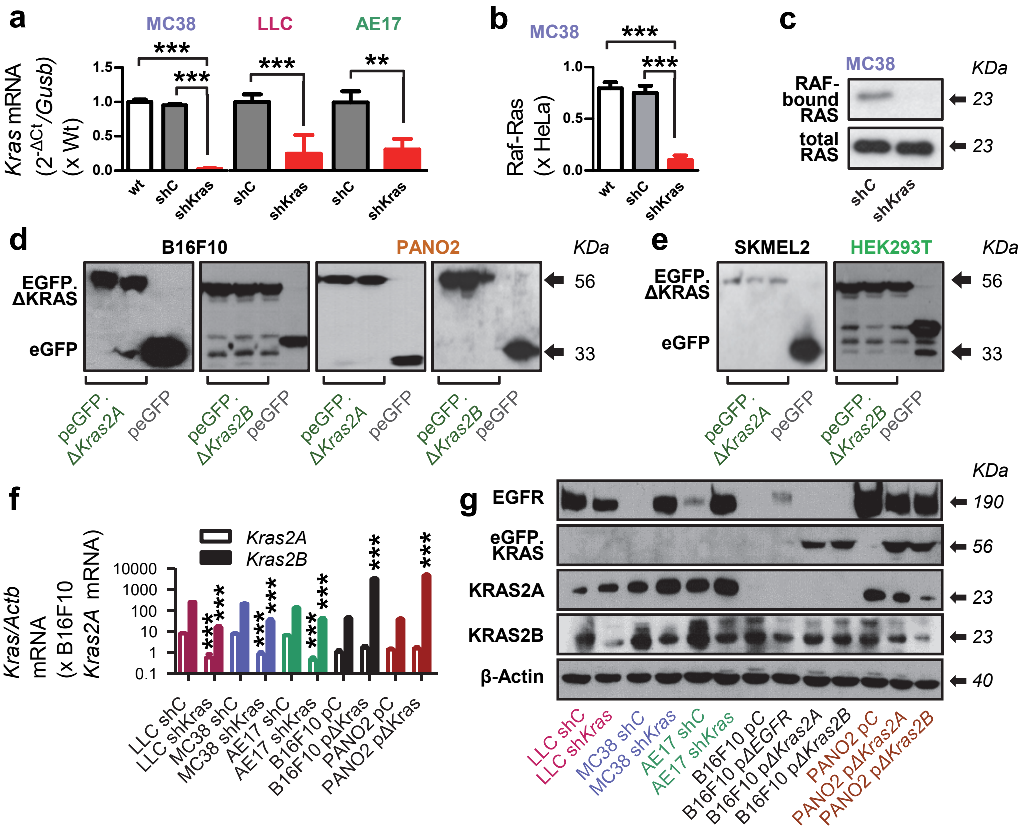
a
MC38
LLC
AE17
Kras mRNA (2-ΔCt/Gusb) (x Wt)
wt
shC
shKras
b
MC38
Raf-Ras (x HeLa)
c
MC38
KDa
RAF-bound RAS
total RAS
23
shC
shKras
d
B16F10
PANO2
KDa
EGFP.ΔKRAS
eGFP
56
33
peGFP.ΔKras2A
peGFP
peGFP.ΔKras2B
e
SKMEL2
HEK293T
f
Kras2A
Kras2B
Kras/Actb mRNA (x B16F10 Kras2A mRNA)
LLC shC
LLC shKras
MC38 shC
MC38 shKras
AE17 shC
AE17 shKras
B16F10 pC
B16F10 pΔKras
PANO2 pC
PANO2 pΔKras
g
EGFR
eGFP.KRAS
KRAS2A
KRAS2B
β-Actin
190
40
B16F10 pΔEGFR
B16F10 pΔKras2A
B16F10 pΔKras2B
PANO2 pΔKras2A
PANO2 pΔKras2B

Supplementary Figure 3 | Manipulation of mutant *KRAS* isoform expression. *Kras*-mutant mouse tumour cell lines were stably transfected with anti-mouse random (sh*C*) or *Kras* (sh*Kras*) shRNA pools. *KRAS*-wild-type mouse and human cell lines were stably transfected with in-house cloned eukaryotic expression vectors encoding enhanced green fluorescent protein (*peGFP*), eGFP in-frame with mouse mutant *Kras*^{G12C} transcript variant A cloned from LLC total RNA (p*eGFP.ΔKras2A*), or eGFP in-frame with mouse mutant *Kras*^{G12C} transcript variant B cloned from LLC total RNA (p*eGFP.ΔKras or* p*eGFP.ΔKras2B*). **a,** Total *Kras* mRNA expression by qPCR relative to *Gusb* control (n = 5-6/group). **b,** Cytoplasmic RAS-activity as determined by binding of Raf-1-RBD coated agarose beads ELISA, relative to HeLa cytoplasmic extracts (n = 5/group). **c,** Cytoplasmic RAS-activity as determined by binding of Raf-1RBD coated agarose beads immunoblot relative to total cytoplasmic RAS. **d, e,** Anti-eGFP immunoblots. **f,** *Kras* isoform mRNA expression by qPCR relative to β-actin (*Actb)* control (n = 5/group). **g,** Anti-EGFR, eGFP, and KRAS isoform immunoblots relative to β-actin loading control. Data are presented as mean ± SD. ** and ***: $P < 0.01$ and $P < 0.001$ for the indicated comparisons (a and b) and for comparison with the respective parental cell line by Student's t-test (a), and one (a and b)- or two (f)-way ANOVA with Bonferroni post-tests. LLC, *C57BL/6* Lewis lung carcinoma; MC38, *C57BL/6* colon adenocarcinoma; AE17, *C57BL/6* malignant pleural mesothelioma; B16F10, *C57BL/6* malignant skin melanoma; PANO2, *C57BL/6* pancreatic adenocarcinoma; FULA, *FVB* urethane-induced lung adenocarcinoma; CULA, *C57BL/6* urethane-induced lung adenocarcinoma; A549, human lung adenocarcinoma; LTP A549, long-term passaged A549 cells having lost the Y chromosome; SKMEL2, human malignant skin melanoma; HT-29, human colon adenocarcinoma; HEK293T, human embryonic kidney cells.

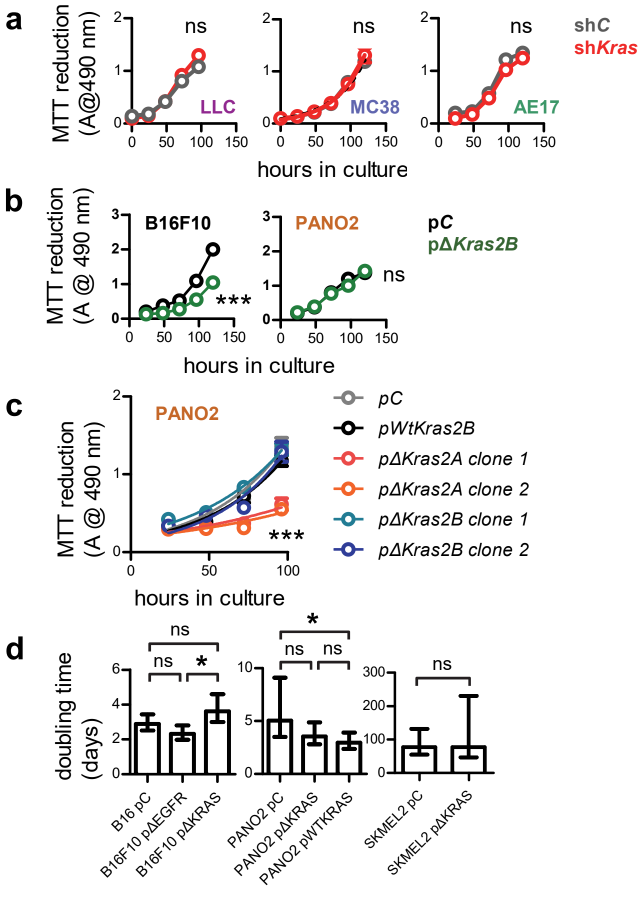

a
MTT reduction (A@490 nm)
ns
LLC
MC38
AE17
shC
shKras
hours in culture
b
MTT reduction (A @ 490 nm)
B16F10
PANO2
pC
pΔKras2B
***
ns
hours in culture
c
MTT reduction (A @ 490 nm)
PANO2
pC
pWtKras2B
pΔKras2A clone 1
pΔKras2A clone 2
pΔKras2B clone 1
pΔKras2B clone 2
***
hours in culture
d
doubling time (days)
ns
*
B16 pC
B16F10 pΔEGFR
B16F10 pΔKRAS
PANO2 pC
PANO2 pΔKRAS
PANO2 pWTKRAS
SKMEL2 pC
SKMEL2 pΔKRAS

Supplementary Figure 4 | Effect of manipulation of mutant *KRAS* isoform expression on tumour cell proliferation. *Kras*-mutant mouse tumour cell lines were stably transfected with anti-mouse random (sh*C*) or *Kras* (sh*Kras*) shRNA pools. KRAS-wild-type mouse and human cell lines were stably transfected with in-house cloned eukaryotic expression vectors encoding enhanced green fluorescent protein (*peGFP*), eGFP in-frame with mouse mutant $Kras^{G12C}$ transcript variant A cloned from LLC total RNA (p*eGFP.ΔKras2A*), or eGFP in-frame with mouse mutant $Kras^{G12C}$ transcript variant B cloned from LLC total RNA (p*eGFP.ΔKras or* p*eGFP.ΔKras2B*). 3 x 10^3 control or *KRAS*-manipulated tumour cells were placed in DMEM-containing 96-well culture dishes (n = 5/group) and MTT reduction was monitored longitudinally. **a-c,** Raw MTT absorbance data presented as mean ± SD. ns and ***: $P > 0.05$ and $P < 0.001$ for comparison with the respective control vector-transfected cell line by two-way ANOVA with Bonferroni post-tests. **d,** Doubling time data calculated by least-squares fit non-linear regression presented as mean ± 95% confidence interval. ns and *: $P > 0.05$ and $P < 0.05$ for the indicated comparisons by one-way ANOVA with Bonferroni post-tests. LLC, *C57BL/6* Lewis lung carcinoma; MC38, *C57BL/6* colon adenocarcinoma; AE17, *C57BL/6* malignant pleural mesothelioma; B16F10, *C57BL/6* malignant skin melanoma; PANO2, *C57BL/6* pancreatic adenocarcinoma; SKMEL2, human malignant skin melanoma.

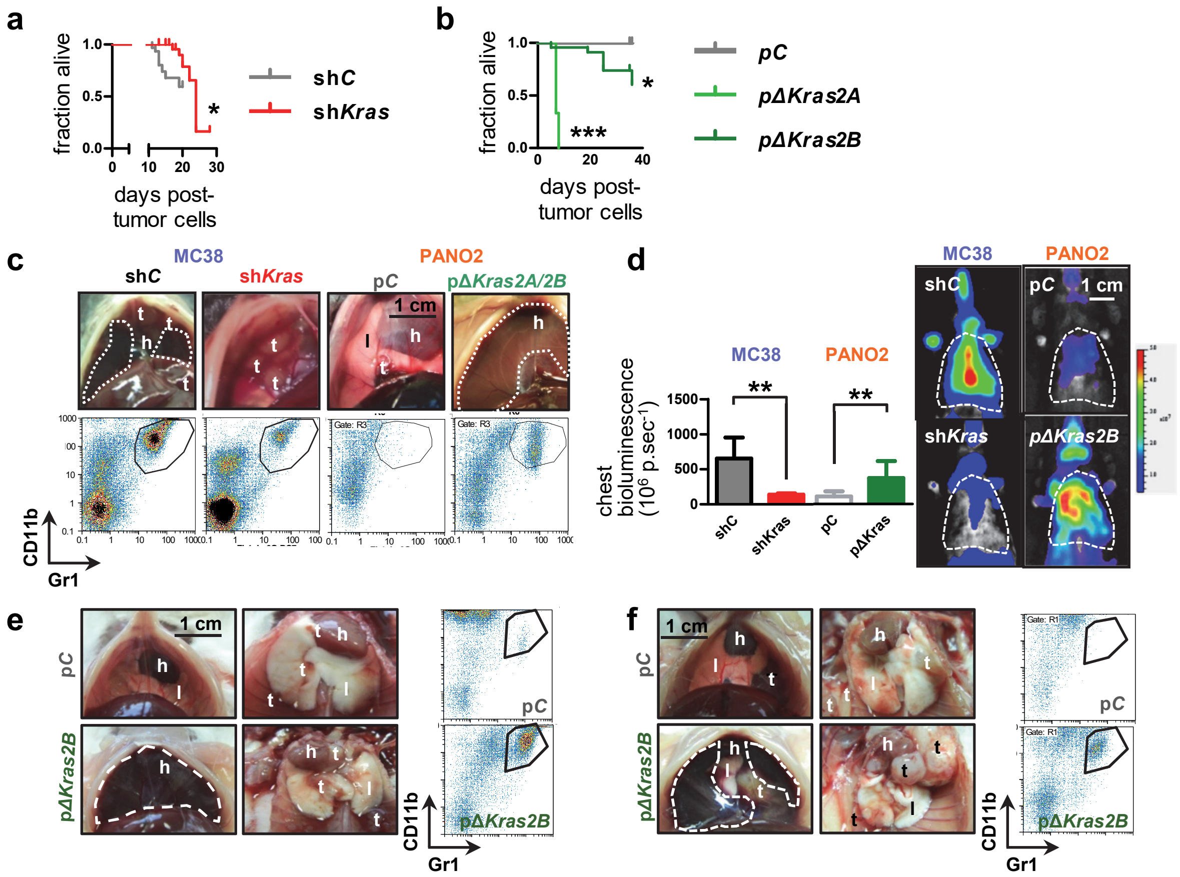
a
fraction alive
days post-tumor cells
shC
shKras
b
pC
pΔKras2A
pΔKras2B
c
MC38
PANO2
shC
shKras
pC
pΔKras2A/2B
1 cm
CD11b
Gr1
d
chest bioluminescence (10^6 p.sec^-1)
shC
shKras
pC
pΔKras
e
pC
pΔKras2B
f

Supplementary Figure 5 | Mutant *KRAS* in malignant pleural effusion development. a, Kaplan-Meier plots of survival of *C57BL/6* mice that received 1.5 x 10^5 intrapleural LLC, MC38, or AE17 cells (all *Kras* mutant) stably expressing random (sh*C*; *n* = 40; grey line) or anti-*Kras* (sh*Kras*; *n* = 35; red line) shRNA. **b,** Kaplan-Meier plots of survival of *C57BL/6* mice that received 1.5 x 10^5 intrapleural PANO2 cells (*Kras* wild-type) stably expressing empty (p*C*; *n* = 8; grey line) or mutant *Kras* isoform 2A (p*ΔKras2A*; *n* = 12; light green line) or 2B (p*ΔKras2B*; *n* = 23; dark green line) expression vectors. **a, b,** * and ***: $P < 0.05$ and $P < 0.001$ for comparison with parental lines by log-rank test. **c,** Representative images (top) of effusions (dashed lines), pleural tumours (t), hearts (h), and lungs (l), and representative dotplots (bottom) of CD11b+Gr1+ cells (polygon gates) from selected experiments described in Fig. 2. **d,** Representative bioluminescent images and data summary of chest light emission (*n* = 5-7/group) of total body-irradiated *C57BL/6* chimeras transplanted with luminescent bone marrow from *CAG.Luc.eGFP* donors at day 14 after pleural injections of the indicated tumour cells. Data are presented as mean ± SD. **: $P < 0.01$ for the indicated comparisons by by Student's t-test. **e, f,** Representative images of malignant pleural disease induced by SKMEL2 (e) and HEK293T (f) cells bearing wild-type (*Wt*) *KRAS* stably expressing empty (p*C*) or mutant *Kras* isoform 2B (p*ΔKras2B*) expression vectors. Shown are representative images of effusions (dashed lines), pleural tumours (t), hearts (h), and lungs (l) and representative dotplots and gating strategy for quantification of CD11b+Gr1+ cells. *Δ*, mutant. LLC, *C57BL/6* Lewis lung carcinoma; MC38, *C57BL/6* colon adenocarcinoma; AE17, *C57BL/6* malignant pleural mesothelioma; B16F10, *C57BL/6* malignant skin melanoma; PANO2, *C57BL/6* pancreatic adenocarcinoma.

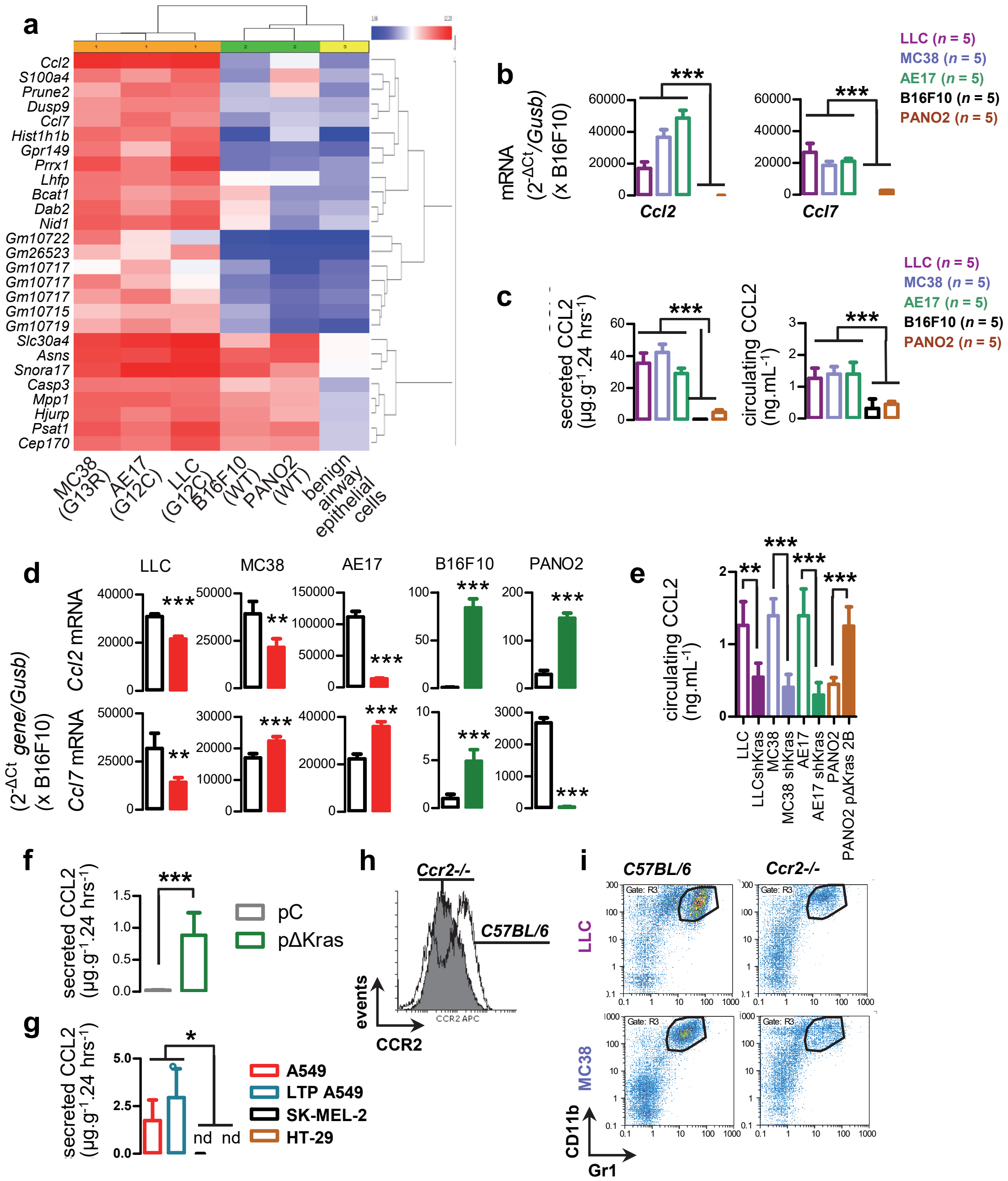
a
Ccl2
S100a4
Prune2
Dusp9
Ccl7
Hist1h1b
Gpr149
Prrx1
Lhfp
Bcat1
Dab2
Nid1
Gm10722
Gm26523
Gm10717
Gm10717
Gm10717
Gm10715
Gm10719
Slc30a4
Asns
Snora17
Casp3
Mpp1
Hjurp
Psat1
Cep170
MC38 (G13R)
AE17 (G12C)
LLC (G12C)
B16F10 (WT)
PANO2 (WT)
benign airway epithelial cells
b
mRNA ($2^{-\Delta Ct}$/Gusb) (x B16F10)
Ccl2
Ccl7
LLC (n = 5)
MC38 (n = 5)
AE17 (n = 5)
B16F10 (n = 5)
PANO2 (n = 5)
c
secreted CCL2 ($\mu g.g^{-1}.24\ hrs^{-1}$)
circulating CCL2 ($ng.mL^{-1}$)
d
LLC
MC38
AE17
B16F10
PANO2
Ccl2 mRNA
Ccl7 mRNA
($2^{-\Delta Ct}$ gene/Gusb) (x B16F10)
e
circulating CCL2 ($ng.mL^{-1}$)
LLC
LLCshKras
MC38
MC38 shKras
AE17
AE17 shKras
PANO2
PANO2 pΔKras 2B
f
secreted CCL2 ($\mu g.g^{-1}.24\ hrs^{-1}$)
pC
pΔKras
g
secreted CCL2 ($\mu g.g^{-1}.24\ hrs^{-1}$)
nd
nd
A549
LTP A549
SK-MEL-2
HT-29
h
Ccr2-/-
C57BL/6
events
CCR2
i
C57BL/6
Ccr2-/-
LLC
MC38
Gate: R3
CD11b
Gr1

Supplementary Figure 6 | Mutant *KRAS* signals via CCL2/CCR2 to recruit CD11b+Gr1+ myeloid cells to the pleural space. a, Comparative analysis of global gene expression of five *C57BL/6* mouse-derived tumour cell lines with defined *Kras* mutation status by Affymetrix mouse gene ST2.0. Shown is unsupervised clustering of cell lines by differentially expressed genes (*Δ*GE) comprising the mutant Kras signature of 25 genes described in Fig. 3a and Table 3. **b,** *Ccl2* and *Ccl7* mRNA expression by five *C57BL/6* mouse-derived tumour cell lines. **c,** Enhanced CCL2 elaboration of *Kras*-mutant murine cell lines in vitro by ELISA (n = 6/group; left) and increased serum CCL2 levels of mice with MPEs induced by *Kras*-mutant murine cell lines *in vivo* by ELISA (n = 6/group; right). (b, c) Colour codes are as in Extended Data Fig. 1. **d,** *Ccl2* and *Ccl7* mRNA expression by parental (white bars: cells stably expressing random shRNA or control overexpression vector) and *Kras*-modulated (red bars: cells stably expressing anti-*Kras*-specific shRNA; green bars: cells stably expressing vector encoding mutant mouse $Kras^{G12C}$ isoform B) murine cell lines from Fig. 2 relative to *Gusb* by qPCR showing transcriptional control of *Ccl2* by mutant *Kras* (n = 5/group). **e,** Serum CCL2 levels of mice bearing pleural tumours with or without MPE induced by parental and *Kras*-modulated murine cell lines from Fig. 2 by ELISA (n = 6/group). **f,** CCL2 secretion of HEK293T cells stably expressing empty (p*C*) or mutant *Kras* isoform 2B (p*ΔKras*2B) expression vectors, assessed by ELISA (n = 6/group). **g,** Enhanced CCL2-elaboration of *KRAS*-mutant human cell lines by ELISA (n = 6/group). **h,** *Ccr2-/-* and *C57BL/6* control mice received intrapleural injections of three different tumour cell lines, as described in Fig. 3c. Shown are representative histograms of pleural cell CCR2 expression by flow cytometry (n = 7/group), **i,** Representative dotplots of CD11b+Gr1+ cells (polygon gates) from experiment in (h).Data are presented as mean ± SD. *, **, and ***: P < 0.05, P < 0.01, and P < 0.001 for comparison with parental lines (d-f) or between any KRAS wild-type and any KRAS-mutant cell line (b, c, g) by Student's t-test (d-f) or one-way ANOVA with Bonferroni post-tests (b, c, g). CCL, C-C-motif chemokine ligand; CCR, C-C-motif chemokine receptor; *Wt*, wild-type; *Δ*, mutant. LLC, *C57BL/6* Lewis lung carcinoma; MC38, *C57BL/6* colon adenocarcinoma; AE17, *C57BL/6* malignant pleural mesothelioma; B16F10, *C57BL/6* malignant skin melanoma; PANO2, *C57BL/6* pancreatic adenocarcinoma; A549, human lung adenocarcinoma; LTP A549, long-term passaged A549 cells having lost the Y chromosome; SKMEL2, human malignant skin melanoma; HT-29, human colon adenocarcinoma.

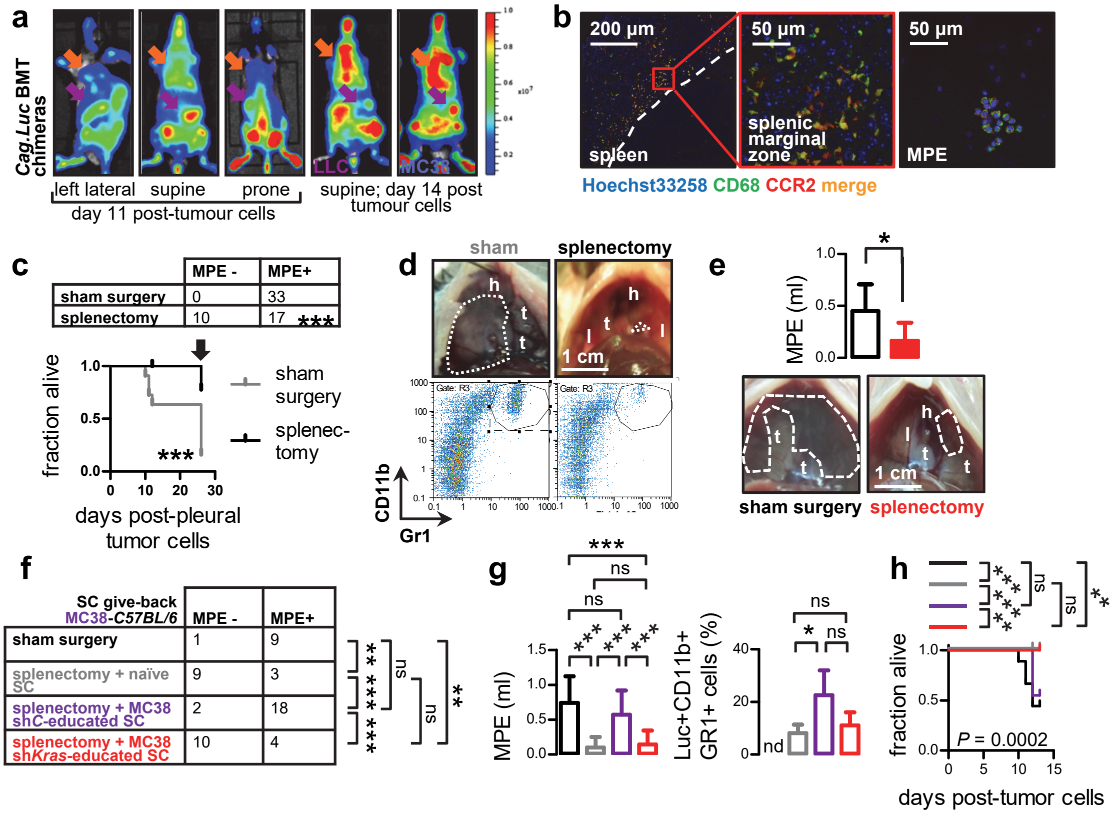
a
Cag.Luc BMT chimeras
left lateral
supine
prone
day 11 post-tumour cells
LLC
MC38
supine; day 14 post tumour cells
b
200 µm
50 µm
50 µm
spleen
splenic marginal zone
MPE
Hoechst33258 CD68 CCR2 merge
c
MPE -
MPE+
sham surgery 0 33
splenectomy 10 17 ***
fraction alive
sham surgery
splenec-tomy
***
days post-pleural tumor cells
d
sham
splenectomy
h
t
l
1 cm
CD11b
Gr1
Gate: R3
e
MPE (ml)
*
sham surgery
splenectomy
f
SC give-back MC38-C57BL/6
MPE -
MPE+
sham surgery 1 9
splenectomy + naïve SC 9 3
splenectomy + MC38 shC-educated SC 2 18
splenectomy + MC38 shKras-educated SC 10 4
***
**
ns
g
MPE (ml)
Luc+CD11b+ GR1+ cells (%)
nd
ns
*
***
h
fraction alive
P = 0.0002
days post-tumor cells

Supplementary Figure 7 | CD11b+Gr1+ myeloid cells traffic to the pleural space via the spleen to promote malignant pleural effusion development. a, Representative whole-body bioluminescent images of mice from experiment described in Supplementary Fig. 1f showing sequential increases of the bone marrow cell-emitted signal in the left subphrenic (magenda arrows) and the thoracic (orange arrows) areas. **b,** Immunofluorescent detection of CCR2 and CD68 in spleens and MPE cells of experimental mice from Fig. 1 localized myeloid cells to the marginal zone (magnified area). **c, d,** *C57BL/6* mice received sham surgery or splenectomy followed by pleural injections of MC38 or PANO2 cells expressing p*ΔKras2A* or p*ΔKras2B*. Shown are MPE incidence table (*n*) and Kaplan-Meier survival plot (c) and representative images of effusions (dashed lines), pleural tumours (t), hearts (h), and lungs (l), and representative dotplots of CD11b+Gr1+ cells (polygon gates) (d). ***: $P < 0.001$ for comparison with sham surgery by Fischer's exact test (table) or by log-rank test (survival plot). Note that even splenectomized mice harvested at late time-points (black arrow in c) did not have MPE, indicating a prolonged protective effect of the intervention. **e,** *NOD/SCID* mice received sham surgery or splenectomy followed by pleural A549 lung adenocarcinoma cells with endogenous mutant $KRAS^{G12S}$. Shown are data summary of effusion volume (n = 6-9/group) and representative images of effusions (dashed lines), pleural tumours (t), hearts (h), and lungs (l). Data are presented as mean ± SD. *: $P < 0.05$ for the indicated comparison by Student's t-test. **f-h,** *C57BL/6* mice received sham surgery or splenectomy followed by pleural MC38 cells after 14 days. At post-injection day 9, splenectomized animals were reconstituted with three million intravenous splenocytes (SC) from *CAG.Luc.eGFP* donors pre-treated 13 days earlier with pleural saline (naïve SC), control shRNA-expressing MC38 cells (MC38 sh*C*-educated SC), or anti-*Kras* shRNA-expressing MC38 cells (MC38 sh*Kras*-educated SC). Shown are MPE incidence table (f), data summaries of effusion volume (*n* as in incidence table under f) and Luc+CD11b+Gr1+ bone marrow-borne splenocytes (n = 5/group) (g), and Kaplan-Meier survival plot (h). Data are presented as mean ± SD. ns, **, and ***: $P > 0.05$, $P < 0.01$, and $P < 0.001$ for the comparisons indicated by χ^2 and Fischer's exact tests (f), by log-rank test (h), or by one-way ANOVA with Bonferroni post-tests (g). SC, splenocyte; Luc, luciferase. *Wt*, wild-type; *Δ*, mutant; MC38, *C57BL/6* colon adenocarcinoma; PANO2, *C57BL/6* pancreatic adenocarcinoma.

Uncropped blots of Supplementary Figure 1B; dashed lines indicate portion shown in composite Figure

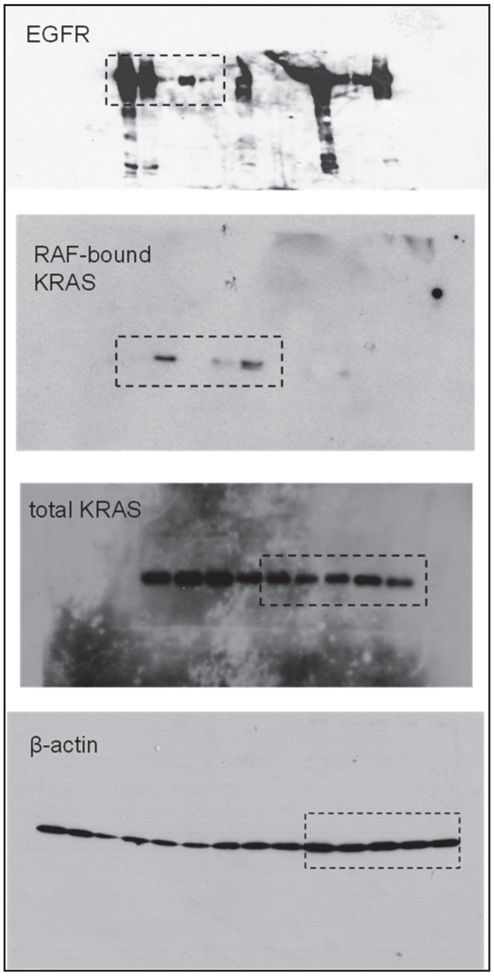

Uncropped blots of Supplementary Figure 1C; dashed lines indicate portion shown in composite Figure

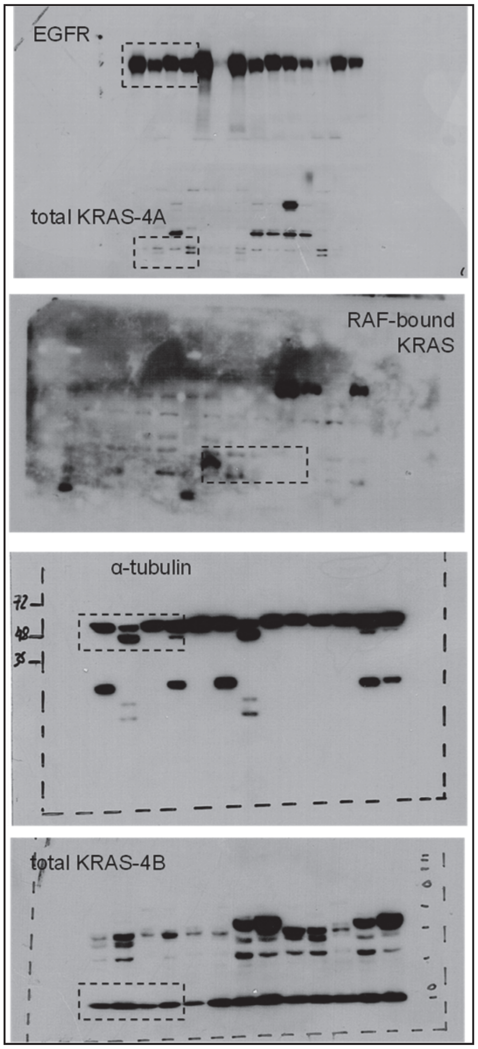

Uncropped blots of Supplementary Figure 1D; dashed lines indicate portion shown in composite Figure

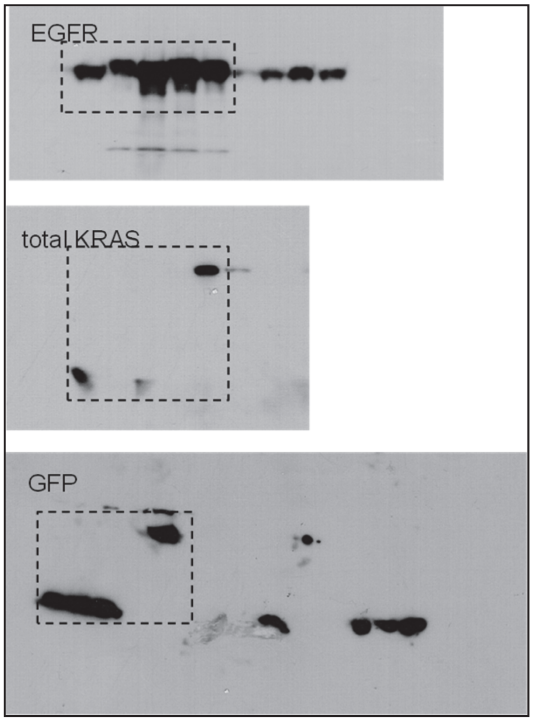

Supplementary Figure 8 | Full blots shown in Supplementary Figure 1.

Uncropped blots of Supplementary Figure 3C; dashed lines indicate portion shown in composite Figure

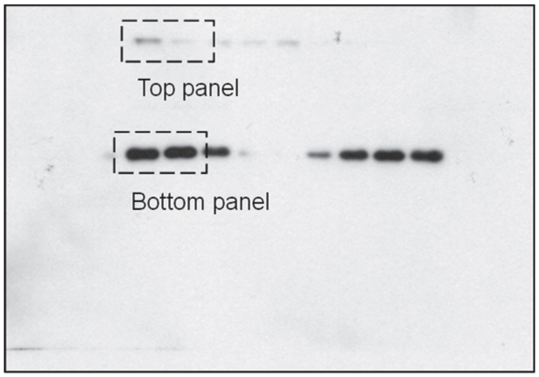

Uncropped blots of Supplementary Figure 3D; dashed lines indicate portion shown in composite Figure

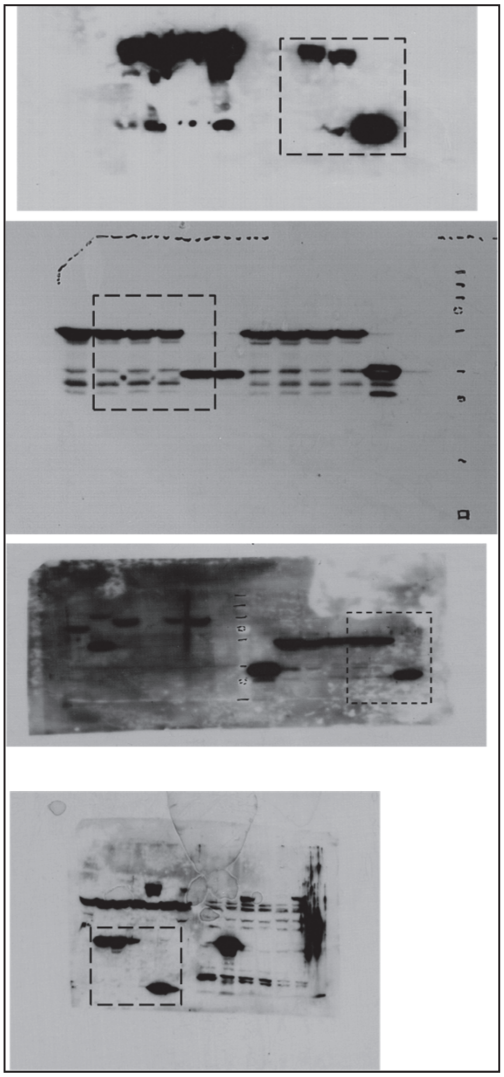

Uncropped blots of Supplementary Figure 3E; dashed lines indicate portion shown in composite Figure

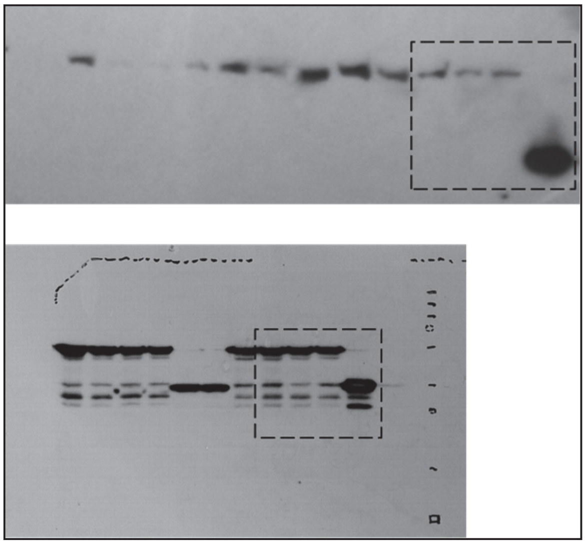

Supplementary Figure 9 | Full blots shown in Supplementary Figures 3c-e.

Uncropped blots of Supplementary Figure 3G; dashed lines indicate portion shown in composite Figure

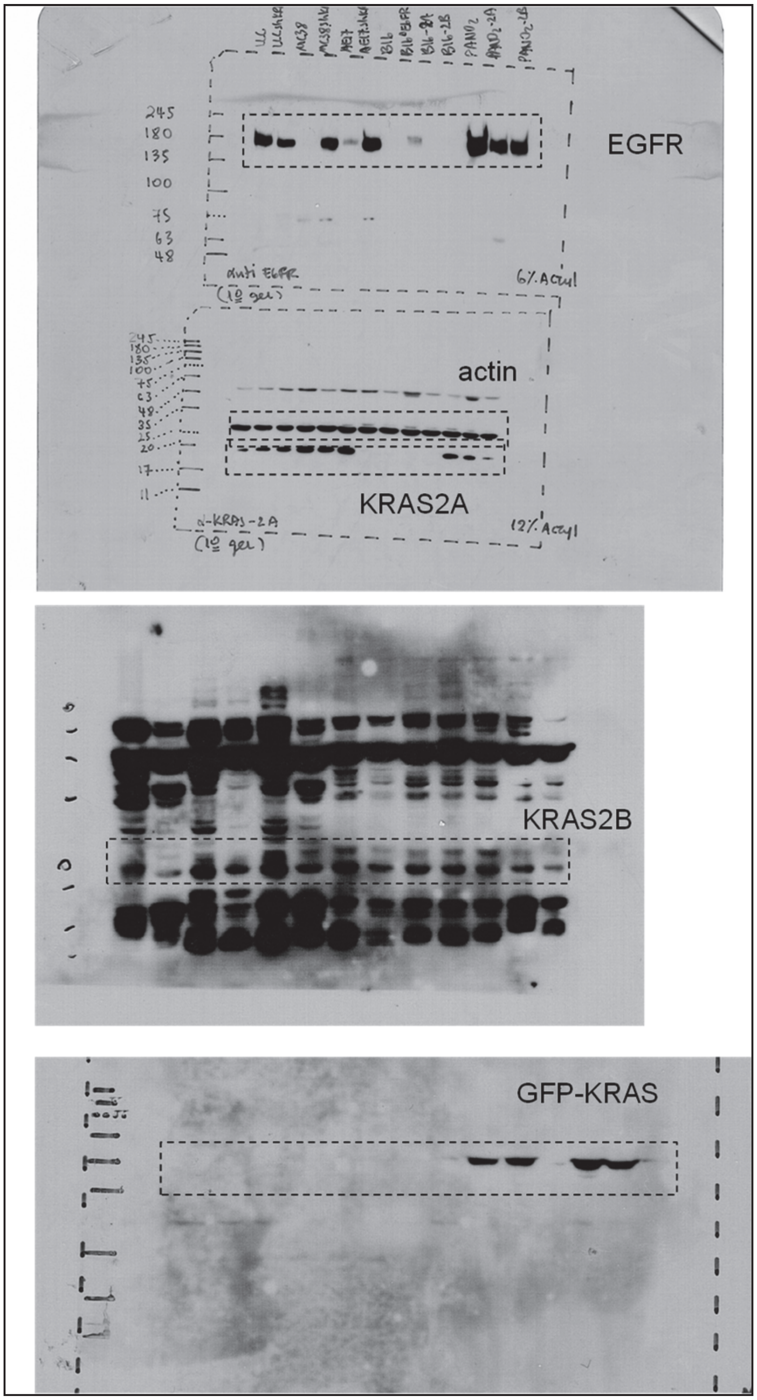

Supplementary Figure 10 | Full blots shown in Supplementary Figure 3g.

Supplementary Table 1 | Oligonucleotide primers used in this study.

Method[a]	Primer	Sequence	Amplicon (bp)
Seq	mKrasF	CCATTTCGGACCCGGAG	905
Seq	mKrasR	CTTTAGTCTCTTCCACAGGCA	
Seq	mEgfrF1	GCCTGATAACTGGACTGACCT	F1/R1 1673
Seq	mEgfrR1	AGAATCAACTCTCGGAACTTTGG	F2R2 2261
Seq	mEgfrF2	CTCCTCTTCTTCCCGCACTG	F2/R 2073
Seq	mEgfrR2	AGAATCTGAGACCTCTGGCTG	1S1/R1 1174
Seq	mEgfrR	GCATAGGTGGCAGACATTATTGG	1S2/R1 672
Seq	mEgfr1S1	ACAACTGCATCCAGTGTGCC	1S1/R2 1759
Seq	mEgfr1S2	GGCCATCAAGGAGTTAAGAG	1S1/R 1571
Seq	mEgfr2S1	AGAGAATCCCTTTGGAGAACC	2S2/R2 1256
Seq	mEgfr2S2	CCACCACTCATGCTGTACAACC	2S2/R 1068
Seq	mEgfr2S3	GTCGTTGGCCTGAACATCAC	2S3/R2 731
			2S3/R 543
Seq	mPik3caF1	ATTCTGACTCCATAAGGCGG	1493
Seq	mPik3caR1	GAACCAATCAAACTCCAACTC	
Seq	mPik3caF2	GCTGAACCCTATTGGTGTTACTG	1958
Seq	mPik3caR2	GCTCAAGTCCTAATGTTGTTCCT	
Seq	mBrafF1	CGCTGTCTTCGGAAATACCA	1551
Seq	mBrafR1	AATTCTTTCCATCATGCCTGACC	
Seq	mBrafF2	GGCGGGTTCCAGAGGTG	2066
Seq	mBrafR2	CACTCCACCGAGATTTCACTG	
Seq	hKRASF	TCCCAGGTGCGGGAGAGAG	722
Seq	hKRASR	GCTAACAGTCTGCATGGAGCAGG	
PCR	*MycoF*	GGGAGCAAACAGGATTAGATACCCT	270
PCR	*MycoR*	TGCACCATCTGTCACTCTGTTAACCTC	
qPCR	mEfgrtv1F	ATCAAAGTTCTGGGTTCGGG	156
qPCR	mEfgrtv1R	CATCACATAGGCTTCGTCAAGG	
qPCR	mEfgrtv2F	AACTGTACCTATGGATGTGCTG	154
qPCR	mEfgrtv2R	GGATTTGGAAGAAACTGGAAGG	
qPCR	mKrasF	CGCCTTGACGATACAGCTAAT	132
qPCR	mKrasR	TGACCTGCTGTGTCGAGAAT	
qPCR	mKras2AF	AGATGTGCCTATGGTCCTGG	144
qPCR	mKras2AR	GCATCCTCCACTCTCTGTCT	
qPCR	mKras2BF	ACTCTGAAGATGTGCCTATGGT	148
qPCR	mKras2BR	TCGTCAACACCCTGTCTTGT	
qPCR	mCcl2F	CTACAAGAGGATCACCAGCAG	145
qPCR	mCcl2R	TTCTGATCTCATTTGGTTCCGA	
qPCR	mCcl7F	CATCCACATGCTGCTATGTCA	126
qPCR	mCcl7R	CTTCCATGCCCTTCTTTGTCT	
qPCR	mGusbF	TTACTTTAAGACGCTGATCACC	165
qPCR	mGusbR	ACCTCCAAATGCCCATAGTC	
qPCR	mGapdhF	TGTGTCCGTCGTGGATCTGA	150
qPCR	mGapdhR	TTGCTGTTGAAGTCGCAGGAG	
CL	mKrasF	GGAGATCTATGACTGAGTATAAACTTGTGGTGG	
CL	mKras2AR	GGGAATTCTTACATTATAACGCATTTTTTAATT	526
CL	mKras2BR	GGGAATTCTCACATAACTGTACACCTTGTCCTT	583

[a]Application: Seq, sequencing; RT, reverse transcriptase-polymerase chain reaction; PCR, DNA polymerase chain reaction; qPCR, quantitative (real-time) PCR; CL, cloning.

Supplementary Table 2 | Antibodies used in this study.

Method[a]	Target	Provider[b]	Catalog #	Dilution/Dose	Conjugate[c]
WIB	KRAS2A	Santa Cruz	sc-522	1:200	-
WIB	KRAS2B	Santa Cruz	sc-521	1:200	-
WIB	β-Actin	Santa Cruz	sc-47778	1:200	-
WIB	eGFP	Santa Cruz	sc-9996	1:200	-
WIB	α-Tubulin	Sigma	T5168	1:4000	-
WIB	EGFR	Abcam	ab52894	1:10000	-
WIB	Goat anti-mouse IgG	Southern Biotech	1030-05	1:8000	HRP
WIB	Goat anti-rabbit IgG	Southern Biotech	4030-05	1:8000	HRP
HIS	CD31	Abcam	ab124432	1:1000	-
HIS	PCNA	Santa Cruz	sc-56	1:50	-
FC	CD45	eBioscience	11-0451	0.1 µg/10^6 cells	FITC
FC	CD11b	eBioscience	12-0112	0.1 µg/10^6 cells	PE
FC	Gr1	BD	552093	0.1 µg/10^6 cells	PerCP-Cy5.5
FC	Gr1	eBioscience	25-5931-82	0.1 µg/10^6 cells	PE-Cy7
FC	Luciferase	Abcam	ab16466	0.5 µg/10^6 cells	
FC, IF	CCR2	R&D	FAB5538A	1 µL/10^6 cells, 1:500	APC
IF	CD68	Serotec	MCA1957	1:500	-
IVN	CCL2	Janssen R&D	C1142	50 mg/Kg intraperitoneally every three days	-

[a]Application: WIB, Western immunoblotting; HIS, histology; FC, flow cytometry; IF, immunofluorescence; IVN, *in vivo* neutralization.

[b]Providers*:* Santa Cruz Biotechnology, San Diego, CA; Sigma Aldrich, Taufkirchen, Germany; Abcam, Cambridge, UK; Southern Biotech, Birmingham, AL; eBioscience, San Diego, CA; AbD Serotec, Kidlington, UK; R&D Systems, Minneapolis, MN; Oncology Discovery Research, Janssen R&D LLC, Spring House, PA.

[c]Conjugates*:* FITC, fluorescein isothiocyanate; PE, phycoerythrin; PerCP, peridinin-chlorophyll protein; Cy, cyanine; APC, allophycocyanin; HRP, horse radish peroxidase.
